# Reproducibility, validation, and failure modes across classical and AI-driven molecular docking

**DOI:** 10.1007/s10822-026-00849-8

**Published:** 2026-06-01

**Authors:** Katiana Simões Kittelson, Allana C. F. Martins, Raquel Possemozer Santos, Gizele Celante, Roberto da Silva Gomes

**Affiliations:** Department of Pharmaceutical Sciences, College of Health and Human Sciences, North Dakota State University, Fargo, ND USA

**Keywords:** Molecular docking, Virtual screening, Scoring functions, Docking validation, Reproducibility benchmarking, Machine learning, Protein-ligand interactions, Computer-aided drug design

## Abstract

Molecular docking is indispensable across computer‑aided discovery. However, its conclusions often hinge more on modeling choices than on software brand or nominal score. In this review, we argue that docking should be treated explicitly as conditional modeling whose interpretability depends on structural provenance, ligand‑state definition, search‑space design, and validation under deployment‑relevant conditions. This framework is intended as a practical reference for evaluating docking rigor across both academic and applied workflows. We highlight recurrent failure modes, cross‑target score comparisons under non‑comparable states, over‑read scores as affinities, under‑modeled solvation/flexibility, and uncritical use of predicted structures, and show how AI both exacerbates and mitigates these risks. We then propose best practices for modern validation (self‑docking as necessary but insufficient; cross‑docking, decoys, apo/predicted structures, and out‑of‑distribution tests as essential complements) and offer a concise FAIR reporting checklist enabling reuse and audit. Looking forward, we contend that the most valuable advances are those that improve deployment‑relevant reliability, pose plausibility, enrich screening, and enhance robustness across receptor uncertainty, rather than tool novelty alone. This review reframes docking success from “obtaining a pose and a score” to earning confidence through transparent workflows and evaluation aligned with real use. In this context, the term “AI-driven” does not imply replacing physics-based docking, but rather expanding the workflow landscape in which classical and machine learning approaches coexist. This review, therefore, treats docking as a unified decision framework spanning both paradigms, with emphasis on how validation, generalization, and reproducibility requirements evolve in AI-assisted workflows.

**Graphical abstract:**

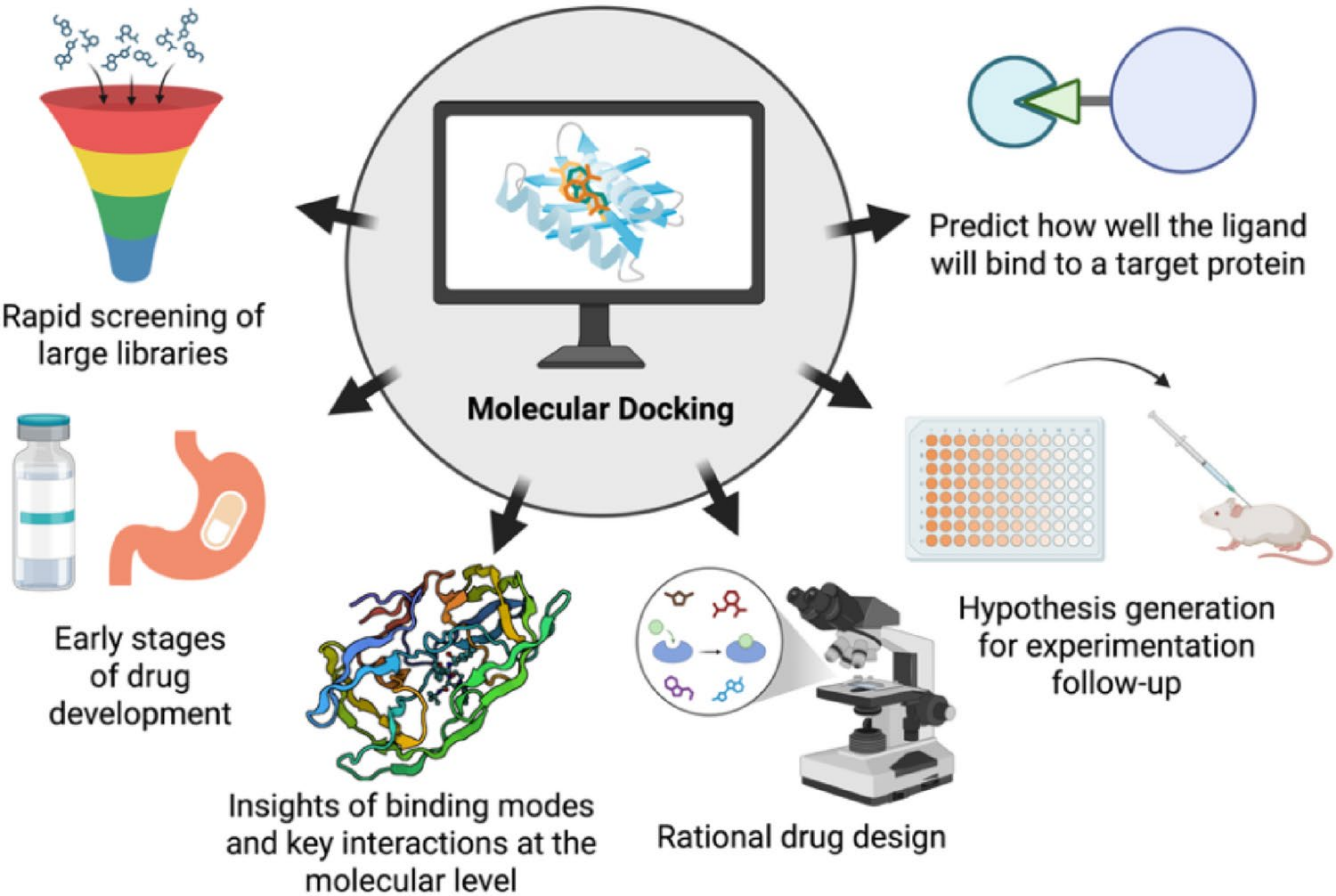

**Supplementary Information:**

The online version contains supplementary material available at 10.1007/s10822-026-00849-8.

## Introduction

Docking is now ubiquitous across discovery workflows, but its conclusions are conditional on modeling choices [1–3]. Molecular docking is a structure-based computational method used to predict how a ligand may bind to a macromolecular target and to estimate plausible binding poses within a defined search space [4–7]. Depending on the system and algorithm, docking protocols may treat the receptor and ligand as rigid, partially flexible, or fully flexible, and they may be performed either in a focused binding site or across a broader protein surface [4, 6, 8]. Because these choices directly influence pose recovery, rank ordering, and interpretability, docking results should be understood as model-dependent hypotheses rather than direct proof of binding.

Molecular docking has already been covered in many broad reviews, such as in the works of Pagadala et al. [9], Mohanty et al. [10], and Paggi et al. [11], Therefore, the purpose of this paper is to focus on the practical decisions that most strongly affect whether docking results are interpretable and reproducible: target and ligand preparation, search-space definition, pose evaluation, modern validation, common failure modes, and transparent reporting. We further position these issues in the context of AI-enabled structure prediction and docking workflows, where stronger benchmarking and leakage-aware evaluation are now essential.

This review defines the minimum conditions under which docking results become interpretable, reproducible, and useful for decision-making. Our focus is on providing a reproducibility-first framework for receptor and ligand preparation, search-space design, pose evaluation, validation, and reporting. Table 1 serves as the organizing framework for the sections that follow, and the later failure modes can be understood as violations of one or more of its components.

## Conceptual foundations for interpretable docking

Molecular docking is fundamentally conditional modeling [12]. A docking result is conditioned by the receptor structure used, the ligand chemical state, the definition of the binding site, the sampling algorithm, and the scoring function [5]. These are first‑class variables determining whether a predicted pose is chemically plausible and whether a score can be meaningfully interpreted [8]. Docking algorithms generate and rank candidate poses using scoring functions that approximate how well a ligand fits within a defined receptor model [8, 13]. We therefore recommend treating preparation choices as reportable parameters to enable audit and reproducibility [5, 14].

Structural sources are a major determinant of docking interpretability. Experimental protein structures, from repositories such as the Protein Data Bank [15], often provide the most reliable starting point. In contrast, predicted structures, such as those generated by AlphaFold [16], can expand target coverage but require additional caution at the pocket level, especially for side-chain placement, local geometry, and conformational realism [17]. Likewise, ligand preparation is not a routine preprocessing step: protonation, tautomer state, stereochemistry, and conformer generation can materially affect downstream pose generation and ranking [12].

Protein flexibility remains one of the central limitations of docking, because many protocols evaluate ligands against a single receptor conformation, whereas ligand recognition often depends on side-chain rearrangement, pocket adaptation, water displacement, and broader conformational changes. Therefore, complementary methods such as molecular dynamics can help address some limitations of static docking, particularly when receptor flexibility or local relaxation is important [18]. However, the central issue for docking remains whether the docking workflow itself has been designed and validated in a way that supports interpretation and reproducibility [5, 6].

The key conceptual point is that docking outputs are conditional on upstream modeling decisions. Structural source selection, protonation-state assignment, search-space definition, and scoring assumptions jointly determine whether a docking result is interpretable. In practice, this means that workflow transparency, validation design, and explicit reporting of modeling assumptions matter at least as much as software choice.

Furthermore, integrating Artificial Intelligence (AI) into these workflows introduces additional sources of variability beyond traditional physics-based assumptions [19]. Because these approaches rely on available training data, risks such as data leakage, overfitting, and poor generalization to unseen data are introduced. Consequently, the interpretation of AI-assisted results must evolve to account for dataset composition and the specific training-test separation used in the model development. Moreover, to maintain scientific rigor, AI-generated results require additional validation, especially when applied to targets that are outside the scope of the training data. Finally, to ensure reproducibility, AI-based frameworks should be thoroughly documented, ensuring that evaluation protocols address and mitigate potential biases [20, 21].

## Practical workflow and modern validation of molecular docking

### A reproducibility‑first framework

A meaningful docking study depends on a transparent workflow that links preparation, docking setup, validation, and pose interpretation. The preparation stage is a major determinant of docking reliability.

**Fig. 1:**
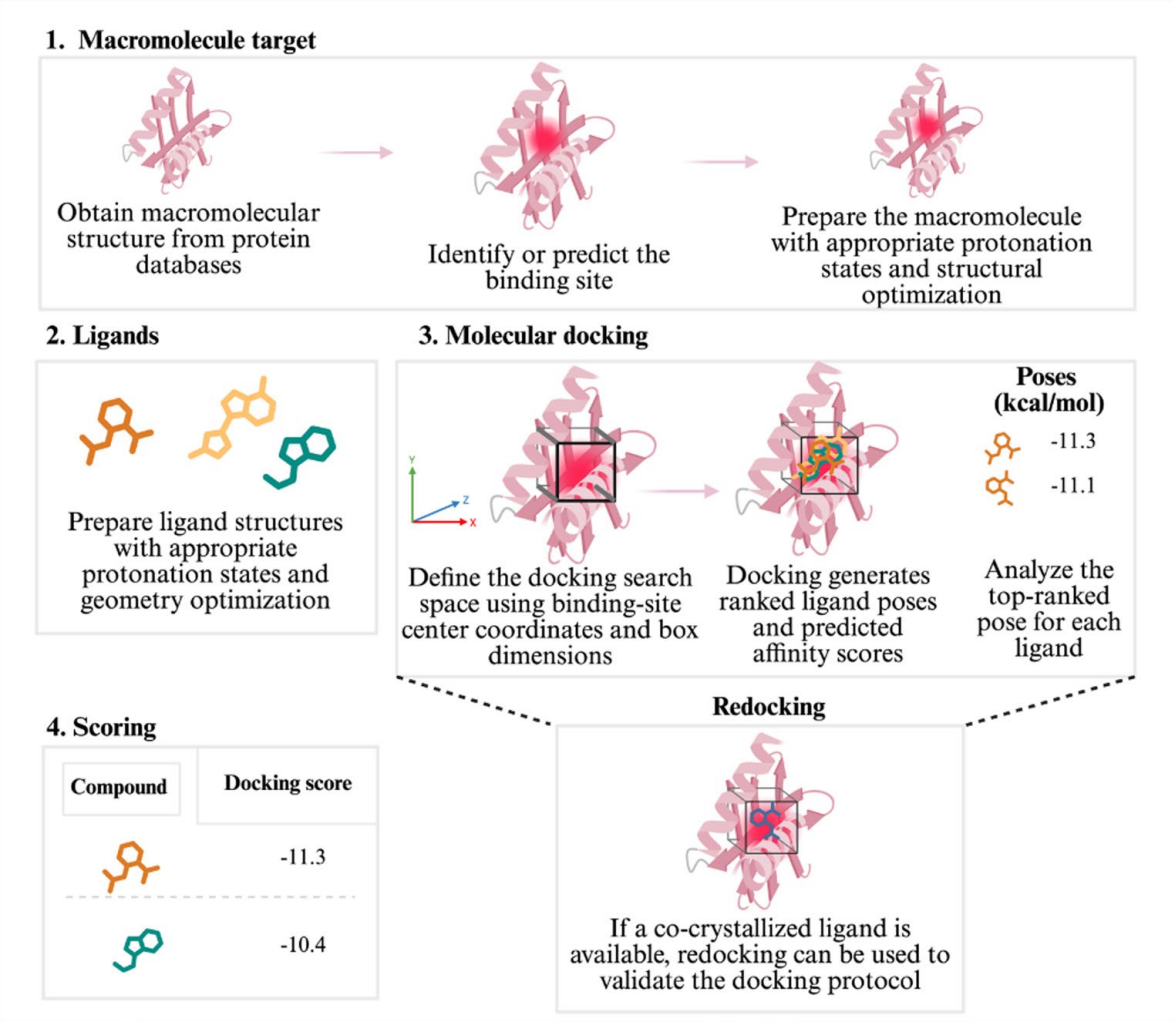
Schematic representation of a reproducibility-first molecular docking workflow highlighting key decision points that determine interpretability. The process begins with macromolecular target preparation, including selecting an appropriate structural source (experimental or predicted), identifying the binding site, preparing the receptor with correct protonation states, and performing structural refinement

For the receptor, users should define the structural source, evaluate missing residues or side chains, and decide, case by case, whether crystallographic waters, cofactors, metals, and co-crystallized ligands should be retained, removed, or used as references for validation. It can also include the reconstruction of missing loops or chains, the resolution of alternate conformations, and the assignment of protonation states. Protein preparation workflows may also involve resolving alternate conformations and treating partial occupancies. Decisions such as selecting a dominant conformation, averaging coordinates, or rebuilding regions using modeling tools can materially alter pocket geometry and interaction patterns and should therefore be explicitly described to ensure reproducibility and correct interpretation. For the ligand, preparation should include chemically sensible protonation and tautomer states, generation of 3D conformers, geometry optimization, and explicit reporting of the software, choice of force field (e.g. GAFF [22] or MMFF [23]), and charge assignment method (e.g. AM1-BCC [24] or RESP) [25]. Some workflows might also require quantum mechanical calculations. These choices strongly affect downstream pose generation and score ranking and, therefore, should be treated as methodological variables rather than routine preprocessing steps. While Fig. 1 provides a visual overview of this workflow, the minimum requirements for interpretability can be formalized into explicit criteria.

To formalize this reproducibility-first plan, Table 1 summarizes the major workflow components that determine docking interpretability, the minimum standard expected for each, the most common red flags, and the scientific risks created when those standards are not met. The remainder of this review uses these components as its core logic: later sections show how they inform minimum practice, how they fail in specific use contexts, and how violations of them produce recurrent interpretive errors.

The docking stage requires explicit definition of the search strategy, as parameters such as grid placement, search-space size, and sampling exhaustiveness have been shown to directly influence pose recovery and virtual screening performance. For example, systematic benchmarking studies demonstrate that inappropriate grid definition or overly large search spaces reduce enrichment and increase false positives by introducing non-physical binding modes [26–28]. Likewise, sampling parameters and exhaustiveness control the ability of docking algorithms to adequately explore conformational space, with insufficient sampling leading to failure in reproducing known binding poses [29, 30]. Therefore, software name, version, and key parameters should be reported clearly so that results can be interpreted and reproduced.

Structural constraints applied during docking, such as van der Waals (vdW) scaling for soft docking, can be implemented either at the grid preparation stage (modifying receptor-ligand interaction potentials) or at the ligand stage (reducing ligand radii during sampling). These choices are not equivalent, as they influence sampling behavior and pose ranking differently, and should therefore be explicitly reported [31].

**Table 1:** Reproducibility-first framework for interpretable molecular docking

Workflow component	Minimum standard	Red flag	Interpretation risk
Receptor selection	Use an experimental structure or a predicted structure validated at the pocket level (side-chain realism, local geometry)	Blind use of predicted structures without pocket validation	False binding modes and misleading interactions
Receptor preparation	Report provenance, edits, missing residues, waters, cofactors, and ligands	Unreported structural edits or arbitrary removal of key elements	Distorted binding-site chemistry and non-reproducibility
Ligand-state definition	Enumerate protonation states, tautomers, stereoisomers, and conformers	Single assumed ligand state without justification	Rank instability and irreproducible results
Search-space definition	Define grid center, dimensions, and biological rationale	Oversized or convenience-based search space	Artificial pose diversity and poor interpretability
Sampling strategy	Report engine, version, exhaustiveness, and number of poses	Default parameters without disclosure	Hidden parameter dependence and irreproducibility
Pose evaluation	Assess steric fit, interaction plausibility, and chemical realism	Lowest-score selection without inspection	Chemically implausible poses and overinterpretation
Validation design	Use cross-docking, decoys, apo/predicted structures, and OOD tests	Self-docking only	Overfitting and inflated confidence
Benchmark splitting / AI evaluation	Use leakage-resistant dataset splitting	Overlap between training and testing sets	False generalization and inflated performance
Score interpretation	Use scores only for within-protocol ranking	Cross-target or cross-protocol comparison	False SAR and misleading prioritization
Mechanistic inference	Support with validation and orthogonal evidence	Claims based only on pose and score	Overstated biological conclusions
Reproducibility/reporting	Report all inputs, parameters, and deposit key artifacts	Missing parameters or structures	Inability to reproduce or audit results

*Minimum standards are proposed for the major workflow components that determine whether docking outputs can be interpreted as credible, reproducible hypotheses. Red flags indicate common shortcuts or reporting failures that weaken interpretability, while the final column identifies the downstream scientific risk created by each deficiency

Structural constraints used during docking can take multiple forms, including distance constraints (enforcing proximity between ligand and key residues), interaction constraints (hydrogen bonds, metal coordination, or π–π interactions), pharmacophore constraints (requiring predefined feature matching), and positional restraints (restricting ligand placement within subregions of the binding site). While these approaches can improve pose recovery in well-characterized systems, they introduce prior assumptions that bias sampling and may artificially inflate apparent performance if not explicitly reported and validated [31].

Additionally, the analysis stage should extend beyond selecting the lowest-scoring pose. Studies have shown weak, target-dependent correlations between docking scores and experimental binding affinities, as well as frequent failures in ranking true binders across datasets [6]. Therefore, choosing poses by score without checking structure may result in chemically implausible interpretations.

Investigators should assess pose quality using objective criteria, including the reproduction of experimentally observed binding modes, the recovery of key interaction motifs, and consistency with known structure-activity relationships. In this context, validation strategies such as cross-docking, decoy-based benchmarking, and retrospective virtual screening are essential to assess whether a docking protocol can discriminate true binders from non-binders [32]. In contrast, mechanistic conclusions should rely on convergent evidence from pose inspection, validation performance, and, where possible, orthogonal data.

Reproducibility remains a critical issue in molecular docking. Minimum reporting to enable audit and reuse: receptor provenance/edits; ligand‑state enumeration; engine/version; search‑space coordinates; sampling settings; validation suite; plausibility criteria; and artifacts (prepared structures, grids, pose files).

Best-practice minimum for interpretable docking. At a minimum, a defensible docking study should: (i) justify receptor-state selection; (ii) enumerate chemically plausible ligand states; (iii) define and report search-space coordinates and rationale; (iv) disclose engine, version, and sampling settings; (v) inspect pose plausibility beyond score alone; (vi) validate beyond self-docking RMSD; and (vii) report sufficient inputs and parameters to permit audit and reuse.

### Modern validation of docking studies

Modern validation requires complementary tests: (a) *Self‑docking*: a necessary but limited sanity check; tests recovery in a familiar context; (b) *Cross‑docking*: evaluates robustness to receptor structural variability. (c) *Decoy/enrichment tests*: measure discrimination between binders and non‑binders. (d) *Apo or predicted‑structure docking*: tests deployment realism when bound conformations are unavailable. (e) *Out‑of‑distribution (OOD) evaluation*: ensures resistance to benchmark leakage and pocket similarity; and (f) *Plausibility checks*: screens poses for steric, geometric, and chemical realism beyond RMSD.

RMSD-based comparison to the co-crystallized ligand can therefore provide an initial measure of pose recovery. However, successful self-docking mainly reflects performance in a familiar structural context and should be interpreted as necessary but not sufficient evidence of protocol reliability.

In classical settings, pose recovery is assessed by comparing the docked ligand with a previously resolved co-crystallized reference structure, which makes RMSD a useful initial benchmark [33–35]. This is the evaluation for self-docking, with RMSD considered acceptable when values are below ~ 2 Å for pose recovery. Nevertheless, this metric reflects a familiar structural context and does not guarantee generalization.

More demanding validation strategies include cross-docking and decoy-based benchmarking [36]. Cross-docking tests whether a ligand can be placed meaningfully into related but non-identical receptor structures, thereby probing robustness to structural variation rather than performance in a single memorized complex [37]. It is typically reported as the percentage of successful pose recoveries under an RMSD threshold. Furthermore, decoy-based evaluation addresses a different question: whether a workflow can distinguish plausible binders from non-binders [38] or unrealistic poses [39]. They use metrics such as the area under the receiver operating characteristic curve (AUC-ROC) and enrichment factors. These complementary metrics provide a stronger assessment of screening utility than self-docking alone [6].

These validation modes should not be treated as interchangeable because each tests a different failure point in the workflow, summarized in Table 1. Self-docking tests protocol consistency in a familiar structural context; cross-docking tests robustness to receptor variation; decoy benchmarking tests screening discrimination; docking into apo-like or predicted structures tests deployment realism; and plausibility checks test whether an apparently acceptable pose is chemically meaningful.

Recent benchmarking trends indicate a growing emphasis on generalization tests under more realistic conditions, including docking into apo-like or predicted structures and evaluating performance across structurally diverse receptor conformations [6, 36, 37]. In AI-assisted workflows, it extends to assessing performance under distribution shift conditions and minimizing dataset leakage [12]. These scenarios better reflect practical use cases, where the crystallographic bound structure may be unavailable and where strong performance on familiar systems may not translate to new targets.

A second major shift is the recognition that acceptable RMSD alone does not guarantee a meaningful pose. Modern evaluation increasingly incorporates leakage-resistant dataset design and plausibility-aware checks, including steric compatibility, chemically sensible geometry, and interaction realism. In this context, readers should understand validation as a combination of pose recovery, screening behavior, out-of-distribution robustness, and physical plausibility. In AI-assisted docking benchmarks, apparent gains may be inflated when structurally related pockets, ligands, or complexes overlap between training, tuning, and evaluation sets; leakage-aware splitting is therefore part of validation, not a secondary implementation detail. In this context, rigorous evaluation requires separation schemes that prevent ligand-, complex-, or pocket-level leakage between training, tuning, and testing, because retrospective gains can otherwise overstate deployment performance.

A minimally reproducible docking report should specify: receptor source and accession/PDB or predicted-model provenance; missing residues or edits introduced during preparation; ligand protonation/tautomer enumeration strategy; docking engine, version, and scoring mode; search-space coordinates and dimensions; exhaustiveness or sampling settings; number of poses retained; validation procedure; and explicit criteria for pose selection and interpretation.

For AI-assisted methods, validation should additionally specify the dataset composition, splitting strategy (ligand-, complex-, or pocket-level), and independence between the training and evaluation sets, as these factors directly determine whether the reported performance reflects true generalization or memorization.

These developments shift the emphasis of docking practice from merely obtaining poses and scores to designing workflows that remain interpretable, reproducible, and defensible under realistic validation settings.

## Stress-testing the reproducibility framework across use contexts

Docking does not fail uniformly across use contexts. The purpose of this section is therefore not to review applications, but to stress-test the reproducibility framework in Table 1 under settings where different sources of uncertainty dominate. In each case, the relevant question is the same: which framework component is most vulnerable, what minimum practice is required, and what kinds of claims should be avoided if that requirement is not met? In this context, Table 2 should be read not as a catalog of platforms, but as a reminder that tool choice always carries modeling assumptions that constrain interpretation [36, 40, 41].

**Table 2:** Representative tools and their implications for docking workflow design and interpretation*

Tool type/class	Representative tool	Core modeling assumption	When valid	Interpretation constraint (what you must NOT claim)	Validation requirement	References
Docking engines with limited receptor flexibility	Autodock Vina, Autodock4	Protein treated as mostly rigid or limited flexibility.	The binding site is pre-organized (co-crystal or well-defined pocket)	Do not interpret a favorable ranking as evidence of a correct binding mode without validation	Cross-docking or orthogonal pose validation and comparison to experimental structures	Trott et al. 2010 [29]; Eberhardt et al. 2021 [5]; Elokely et al. 2013 [42]
High-precision docking (single-structure)	Glide (SP/XP)	Optimized scoring on a single receptor conformation	Systems with known binding mode; high-quality structures	Do not compare scores across targets or receptor states; avoid mechanistic claims without validation	Matched-state benchmarking and pose validation	Shamsian et al. 2023 [8]; Hevener et al. 2009 [35]
Ensemble or flexible docking approaches	GOLD, ensemble docking workflows	Partial receptor flexibility or multiple conformations sampled	Systems with known conformational variability or multiple states	Do not assume completeness of sampling; absence of binding mode ≠ absence of binding	Ensemble sensitivity analysis	Dequeker et al. 2022 [37]; Elokely et al. 2013 [42]
Docking GUIs and workflow wrappers	PyRx, Chimera-based pipelines	Simplified workflows with hidden or default parameters	Educational use, rapid prototyping	Do not assume parameter transparency or reproducibility; avoid publication without explicit parameter disclosure	Full parameter disclosure and reproducibility check	Masocha et al. 2025 [43]; Dallakyan et al. 2015 [44]
Ligand preparation tools	Avogadro, HyperChem, LigPrep	Single or limited ligand state (protonation, tautomer, stereochemistry)	Chemically well-defined ligands with unambiguous states	Do not assume ranking stability across untested ligand states; avoid single-state conclusions	Ligand-state robustness check	Agu et al. 2023 [7]; Jakalian et al. 2002 [24]; Halgren 1996 [23]
Protein preparation tools	Chimera, Protein Preparation Wizard, PDBFixer	Structural refinement defines docking-ready receptor	When starting experimental or predicted structures, requiring cleanup	Do not assume structural correctness without reporting modifications	Documentation of preparation steps and structural validation	Burley et al. 2019 [15]; Mazhibiyeva et al. 2025 [17]
Molecular dynamics (post-docking refinement)	GROMACS, AMBER	Classical force-field dynamics refine static poses	Evaluating local stability of all plausible binding modes	Do not treat MD stability as binding validation; do not infer affinity without free-energy methods	Pose stability analysis plus orthogonal validation beyond MD persistence	Lemkul 2024 [45]; Wang et al. 2004 [22]; Case et al. 2025 [46]
Free energy methods (FEP/FEC)	FEP+, alchemical methods	Accurate relative binding free energy within congeneric series	Small chemical modifications with reliable initial poses	Do not apply to diverse scaffolds; requires correct binding mode and careful setup	Pose correctness and congeneric-series applicability check	King et al. 2021 [47]; Ross et al. 2023 [48]
AI-based structure prediction	AlphaFold, RoseTTAFold	Predicted structures approximate native fold; local errors possible	When experimental structures are unavailable, and pocket confidence is high	Do not assume pocket accuracy; avoid direct docking without local validation	Pocket-level validation	Baek et al. 2021 [49]; Harris et al. 2024 [50]; Abramson et al. 2024 [16]; Mazhibiyeva et al. 2025 [17]
AI-based scoring functions	RF-Score, NNScore, RTMScore	Binding affinity inferred from learned patterns (training data)	Within chemical and structural space similar to training data	Do not assume generalization; risk of dataset leakage and overfitting	Leakage-resistant external benchmarking	Chatterjee et al. 2023 [12]; Liu et al. 2024 [19]; Zhang et al. 2021 [51]
Binding pocket prediction tools	P2Rank, ML pocket detectors	Predicted pockets approximate ligand-binding sites	Proteins with well-defined, conserved pockets	Do not assume predicted pocket is biologically relevant; validate before docking	Pocket relevance validation with structural or experimental support	Ghersi & Sanchez 2009 [4]; Abramson et al. 2024 [16]; Krivák et al. 2018 [52]
Covalent docking frameworks	CovDock, CarsiDock-Cov	Reactive binding modeled via predefined chemistry rules	Systems with known reactive residues and warheads	Do not treat as standard docking; requires mechanism-aware validation	Mechanism-aware validation	Shen et al. 2025 [53]; da Silva et al. 2022 [54]; Scarpino et al. 2018 [55]
Generative/de novo design tools	REINVENT, generative ML	Optimization guided by scoring functions or objectives	Early-stage hypothesis generation	Do not assume synthetic feasibility or binding realism; it requires downstream validation	Synthetic feasibility assessment and experimental follow-up	Blaschke et al. 2020 [56]; Özçelik et al. 2025 [57]

*Representative tools are provided as illustrative examples rather than an exhaustive listing. Software selection should be justified by the biological question, receptor uncertainty, ligand class, and validation objective rather than by popularity or convenience alone

### Drug repurposing and polypharmacology

In drug repurposing and polypharmacology studies, the dominant risk is overinterpretation of scores across non-comparable targets, receptor states, or pocket definitions [14]. Apparent score differences may reflect setup non-equivalence rather than meaningful target preference [58].

*Primary vulnerability in* Table 1 Score interpretation, receptor selection, and search-space definition.

*Minimum practice* Justify receptor-state comparability, apply consistent preparation logic across targets, and restrict interpretation to within-target prioritization unless receptor states and scoring conditions have been deliberately normalized.

*What should not be claimed* Potency-style selectivity or cross-target preference inferred directly from non-comparable docking scores.

### Nutraceuticals and natural compounds

In natural-product and nutraceutical docking, the dominant risk is ligand-state ambiguity. Protonation, tautomerism, stereochemistry, and even the bioactive form may be insufficiently constrained so that ranking differences may reflect preparation choices rather than meaningful target discrimination [7, 59].

*Primary vulnerability in* Table 1 Ligand-state definition and pose evaluation.

*Minimum practice* Enumerate plausible protonation states, tautomers, stereoisomers, and conformers when chemically justified, and state whether the proposed binding hypothesis remains stable across them.

*What should not be claimed* A unique binding mechanism from a single assumed ligand state when the chemically relevant form is uncertain.

### Ion channels, GPCRs, and membrane proteins

In ion channels, GPCRs, and other membrane proteins, the dominant risk is receptor-state mismatch. These systems often populate multiple functional conformations, and a favorable score in one rigid structure may still be inconsistent with the relevant biological state [60, 61].

This challenge is particularly well documented for GPCRs, where inactive and active conformations differ substantially and can lead to conflicting docking interpretations if state selection is not explicitly justified.

*Primary vulnerability in* Table 1 Receptor selection, receptor preparation, and validation design.

*Minimum practice* Justify the functional state selected for docking, consider whether the modeled environment is compatible with the binding hypothesis, and use state-aware or ensemble strategies when structural heterogeneity is likely to matter.

*What should not be claimed* Mechanistically relevant binding from a single rigid structure without support for state relevance.

### Large-scale docking

In large-scale docking, the central question is not whether an individual ligand can be assigned a plausible pose [1, 6, 62], but whether the workflow produces rankings that remain useful under receptor uncertainty and screening noise [63].

*Primary vulnerability in* Table 1 Validation design, benchmark splitting/AI evaluation, and score interpretation.

*Minimum practice* Report enrichment-oriented metrics, examine sensitivity to receptor choice or receptor-state uncertainty, and explicitly account for the downstream cost of false-positive triage.

*What should not be claimed* Mechanistic confidence for every prioritized compound solely because the workflow scales to large libraries.

## Common failure modes in docking interpretation

The most persistent limitations of molecular docking do not arise primarily from software performance, but from systematic interpretive errors. These errors recur across the literature because docking outputs are often treated as direct evidence rather than as conditional hypotheses shaped by modeling assumptions. The failure modes below represent the most common ways in which otherwise technically correct workflows produce misleading conclusions [6, 8, 42].

Each failure mode below corresponds to one or more breakdowns in the reproducibility framework in Table 1, which is why these problems recur even when nominal workflows appear technically correct.

### Score overinterpretation

A central and recurrent error is interpreting docking scores as quantitative measures of binding affinity or as directly comparable metrics across targets, receptor states, or docking protocols [5, 6, 8]. Because scoring functions are calibrated within specific modeling assumptions, differences in score may reflect changes in receptor preparation, search-space definition, or scoring behavior rather than meaningful differences in binding [5, 8].

Minimum practice should therefore treat docking scores strictly as within-protocol ranking tools under comparable conditions. Cross-target or cross-protocol comparisons should only be made when receptor states, search spaces, and scoring regimes have been explicitly normalized. Failure to respect these constraints leads to false structure–activity relationships and misleading prioritization decisions [6, 62].

### Receptor-state under-modeling

Docking results are highly sensitive to the receptor’s structural state [36, 37, 42]. Use of a single rigid conformation, particularly when derived from predicted structures or from a bound state not aligned with the intended mechanism, can produce poses that are internally consistent but biologically irrelevant [16, 36, 50].

Minimum practice should include explicit justification of receptor-state selection, assessment of local pocket realism, and, where appropriate, evaluation across multiple receptor conformations [36]. When structural uncertainty is high, conclusions should be framed as state-dependent hypotheses rather than as general binding claims.

Neglecting receptor-state variability can lead to confident but incorrect binding-mode assignments.

### Ligand-state under-specification

Ligand preparation is a major determinant of docking outcome, yet it is frequently treated as a fixed preprocessing step [5, 8]. Protonation state, tautomeric form, stereochemistry, and conformational ensemble can all materially affect pose generation and ranking [7]. Minimum practice should include enumeration of chemically plausible ligand states and explicit reporting of the preparation strategy. When ranking or pose selection is sensitive to these choices, that dependence should be disclosed.

Using a single arbitrarily selected ligand state can produce unstable rankings and non-reproducible conclusions [7].

### RMSD-only validation

Self-docking with RMSD comparisons to a co-crystallized ligand is often considered sufficient validation [33, 34, 36]. However, successful pose recovery in a familiar structural context primarily demonstrates consistency with that specific system and does not guarantee performance under realistic deployment conditions [33, 37, 38]. Minimum validation should extend beyond RMSD to include cross-docking, enrichment or decoy-based evaluation, and tests under apo-like or predicted structures when relevant [37–39, 41]. These complementary approaches assess robustness, discrimination, and generalization. Reliance on RMSD alone can produce overfitting to familiar complexes and inflate confidence in workflows that have not been tested under deployment-relevant uncertainty [64].

### Neglect of solvation and flexibility

Standard docking protocols often simplify or omit key physical contributions, including explicit solvent effects, water-mediated interactions, and receptor flexibility [42, 65]. While these approximations enable computational efficiency, they can distort interaction patterns and ranking behavior when such effects are mechanistically important [37, 42]. Minimum practice should include awareness of when these factors are likely to influence binding and, where necessary, incorporation of complementary methods such as molecular dynamics, water-aware scoring, or ensemble approaches [42]. Ignoring solvation and flexibility can result in geometrically plausible but physically unrealistic binding hypotheses [37, 42].

### Unsupported mechanistic inference

A frequent downstream error is elevating a docking pose and score into a mechanistic claim without sufficient supporting evidence [64]. A single pose, even if energetically favorable, does not establish binding mode, specificity, or biological activity [64].

Mechanistic conclusions should be supported by convergent evidence, including pose plausibility, validation performance, and, where possible, orthogonal computational or experimental data [53]. Docking alone is best treated as a hypothesis-generating step rather than a definitive mechanistic proof [64]. When this boundary is not respected, docking results can be overinterpreted as evidence of biological function or therapeutic potential.

## Future perspective

These failure modes are not independent; they often arise in combination. Score overinterpretation may coincide with receptor-state mismatch, or RMSD-based validation may mask ligand-state sensitivity. The common thread is that docking outputs are conditional on modeling choices, and errors occur when those conditions are ignored or underreported. Accordingly, rigorous docking practice requires not only technical execution, but explicit recognition of the assumptions that define what a given result can and cannot support.

### Advances that improve pose realism

The value of new docking methods should be judged by whether they reduce known interpretive failures rather than by software novelty alone. Advances are most useful when they improve pose realism under difficult conditions such as reactive chemistry, receptor-state mismatch, or structurally complex binding environments. Accordingly, the developments below are discussed in terms of how they strengthen or complicate rigorous docking practice.

Tool novelty alone should not be taken as evidence of better deployment performance. A tool’s value should be judged by whether it improves pose plausibility, benchmark behavior, and reproducibility under realistic use conditions [64].

Covalent docking illustrates a broader principle of this review: as mechanistic complexity increases, confidence should rely less on nominal docking success and more on mechanism-aware setup and orthogonal support [54]. For reactive ligands, convincing workflows should justify reactive-residue selection, pre-reactive pose plausibility, and the criteria used to distinguish chemically credible solutions from scoring artifacts [53, 54].

Newer covalent-docking frameworks may reduce false positives arising from applying conventional docking assumptions to reactive systems [53]. However, these methods should still be judged by deployment-relevant validation rather than by software novelty alone.

### Advances that improve validation and generalization

AI methods enter docking workflows at distinct levels that carry different assumptions and validation requirements, and rather than removing the core limitations of docking, they shift where errors can enter the workflow. These include: (i) pose generation, where learned models propose binding configurations directly; (ii) scoring or rescoring, where physics-generated poses are ranked using machine-learned functions; (iii) binding-site or pocket prediction, where AI defines the search space; and (iv) end-to-end or co-folding approaches, where protein–ligand complexes are predicted jointly [12]. These categories are not interchangeable, and each introduces different risks of bias, data leakage, and generalization failure.

In AI-assisted docking, additional risks include training-test leakage, hidden dependence on benchmark composition, overfitting to recurring pocket families, and false confidence when predicted structures are used without deployment-relevant evaluation. As a result, AI-era validation should emphasize data-splitting strategy, out-of-distribution assessment, and benchmark designs that reflect real deployment settings rather than only retrospective performance [12].

AI is most useful in docking when it strengthens benchmark design, pocket identification, structure preparation, rescoring, or pose filtering under explicitly defined generalization settings [1, 12, 16]. Predicted structures increase access and risk, but they also increase the importance of pocket-level reliability checks, side-chain realism, and deployment-relevant benchmarking. Structure prediction by AI also includes co-folding approaches, in which protein-ligand or protein-protein complexes are jointly predicted. Emerging frameworks such as Boltz-2 aim to directly infer binding configurations [66]. However, their outputs depend strongly on training data composition and internal confidence metrics. Unlike docking, these methods do not explicitly sample alternative poses within a defined energy landscape, and their confidence scores do not necessarily correspond to thermodynamic stability or binding affinity. As a result, co-folding outputs should be interpreted as structural hypotheses that require validation against experimental data, orthogonal computation, or docking-based plausibility checks rather than as direct evidence of binding mode correctness [66].

Overall, AI methods can be integrated across multiple stages of docking workflows, but their value depends on careful validation, transparent reporting, and explicit consideration of generalization limits. When used carefully, these tools can reduce some longstanding bottlenecks in docking workflows, but they do not eliminate the need for transparent reporting and orthogonal support [12, 67].

### Advances that improve downstream decision quality

Alchemical free-energy methods address a different problem from docking and should be framed accordingly. Docking is most useful for pose generation, triage, and hypothesis formation, whereas FEP/FEC methods become relevant once chemically credible poses or congeneric series have already been established. Their value in this review is therefore not that they replace docking, but that they reduce overextension of docking into affinity-ranking questions it is not designed to answer [48].

Recent methodological improvements have made FEP/FEC workflows more practical. Still, their value here lies in supporting decisions that go beyond the intended scope of docking rather than replacing docking itself [48]. Moreover, specialized software such as FEP + can be applied to chemical modifications, including covalent inhibitors [48]. In this context, the value of alchemical methods lies in refining decisions that docking alone is not intended to resolve.

### Advances that improve reproducibility and reuse

FAIR principles are directly relevant to docking because reproducible conclusions require access not only to final scores or selected poses, but also to the structural inputs, ligand-state definitions, search-space settings, software versions, and filtering logic that produced them. In this context, FAIR practice is not peripheral data management; it is part of the evidentiary chain that allows docking claims to be checked, compared, and reused [48, 68].

For docking studies, FAIR practice should minimally include deposition of receptor identifiers or prepared structures, ligand-state definitions, docking parameters, grid definitions, software versions, pose files, and validation scripts or notebooks sufficient to reproduce the reported ranking and pose-selection logic [69]. In this setting, FAIR compliance is not a peripheral data-management exercise, but part of the evidence chain that allows docking conclusions to be checked, compared, and reused [68]. This is especially important when conclusions depend on choices that are often underreported, such as protonation strategy, search-space definition, pose filtering, or benchmark composition [70].

## Conclusions

The major challenge in molecular docking is no longer access to software, but the ability to generate conclusions that are interpretable, reproducible, and robust to realistic validation settings. Accordingly, docking studies should be evaluated not only by convenience metrics such as self-docking RMSD, but also by the transparency of the workflow, the plausibility of predicted poses, and the extent to which conclusions are supported by benchmark design and orthogonal validation. In that sense, the future of docking lies not simply in newer algorithms, but in more rigorous practice.

Docking should no longer be judged by its ability to produce poses alone, but by whether those poses remain interpretable under explicit modeling assumptions, realistic validation settings, and transparent reporting conditions. In that sense, the future of docking lies not in claiming more from the method, but in defining more rigorously what the method can legitimately support.

## Supplementary Information

Below is the link to the electronic supplementary material.

The supporting information contains a step-by-step workflow for ligand preparation, docking setup, and pose analysis using Avogadro, Discovery Studio, and PyRx. The supporting information should not be interpreted as a universally optimal protocol. Upon acceptance, a public repository will contain representative prepared receptor and ligand files, grid definitions, example docking parameters, output pose files, and reporting templates sufficient to reproduce the tutorial workflow described in this review.

## Data Availability

No datasets were generated or analysed during the current study.

## Abbreviations

ΔG: Binding free energy
ADME: Absorption, distribution, metabolism, and excretion
AI: Artificial intelligence
CADD: Computer-aided drug design
DUD-E: Directory of useful decoys, enhanced
FAIR: Findable, accessible, interoperable, and reusable
FEP: Free energy perturbation
HTS: High-throughput screening
MD: Molecular dynamics
ML: Machine learning
MLSFs: Machine-learning scoring functions
OOD: Out-of-distribution
RMSD: Root mean square deviation
SFs: Scoring functions
