# Supplementary material for: Reproducibility, validation, and failure modes across classical and AI-driven molecular docking
Source: J Comput Aided Mol Des. 2026 Jun 1;40(1):137. doi: 10.1007/s10822-026-00849-8 (PMC13226364; doi:10.1007/s10822-026-00849-8)
Supplement: Supplementary file 1 — The supporting information contains a step-by-step workflow for ligand preparation, docking setup, and pose analysis using Avogadro, Discovery Studio, and PyRx. The supporting information should not be interpreted as a universally optimal protocol. Upon acceptance, a public repository will contain representative prepared receptor and ligand files, grid definitions, example docking parameters, output pose files, and reporting templates sufficient to reproduce the tutorial workflow described in this review. [file 10822_2026_849_MOESM1_ESM.docx]

**Supplementary Information for**

**Reproducibility, Validation, and Failure Modes Across Classical and AI-Driven Molecular Docking**

Katiana Simões Kittelson^#^, Allana C. F. Martins^#^, Raquel Possemozer Santos, Gizele Celante,^*^ Roberto da Silva Gomes^*^

Department of Pharmaceutical Sciences, College of Health and Human Sciences, North Dakota State University, Fargo, ND, United States.

^#^Authors contributed equally

^*^Corresponding author: [roberto.gomes@ndsu.edu](mailto:roberto.gomes@ndsu.edu); [gizele.celante@ndsu.edu](mailto:gizele.celante@ndsu.edu)

**Table of Contents**

1. Protocol for ligand optimization using Avogadro S2

# 2. Protocol for AutoDock Vina Docking using Discovery Studio and PyRx S5

## 3. PyRx docking S7

## 4. Complex visualization on Discovery Studio S9

This tutorial illustrates one practical redocking workflow and should not be interpreted as a universally optimal protocol. Decisions regarding receptor cleanup, protonation, cofactors, conserved waters, and grid definition should be made case by case according to the biological system and validation goal.

To exemplify the protocols, the protein 1HSG with its crystallized ligand from PDB will be used.

**1. Protocol for ligand optimization using Avogadro**

1. Open 2D ligand structure;
2. Avogadro will build a 3D structure from the 2D design;

- *This is the 3D structure of the Ligand_1HSG that will be further used in the re-docking protocol.*


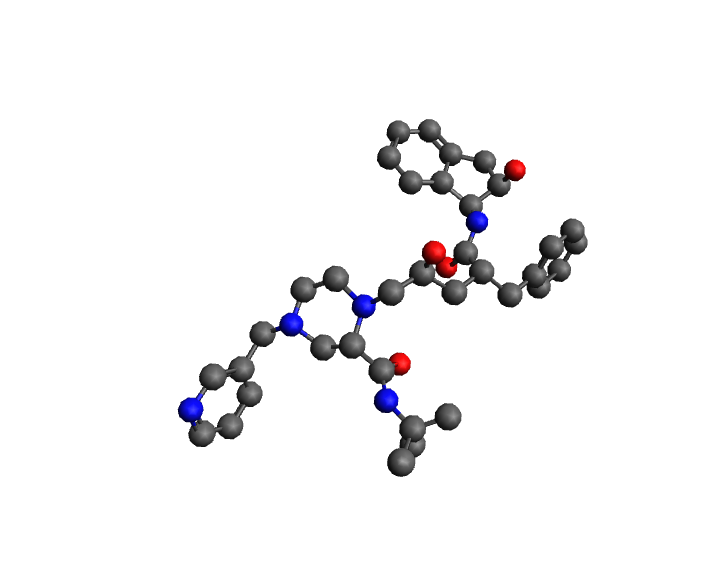


1.
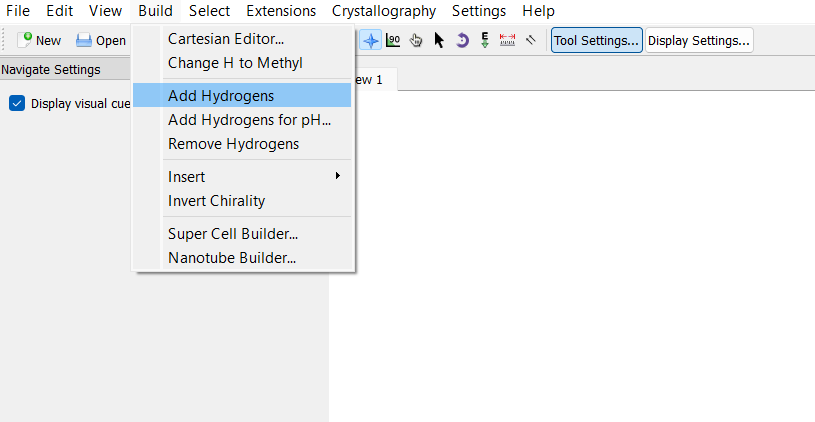
On the main taskbar, select **Build > Add Hydrogens** to add hydrogens to current structure;


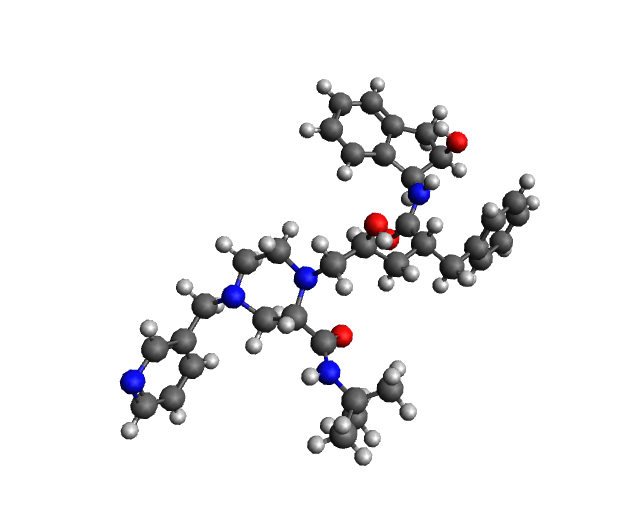


1.
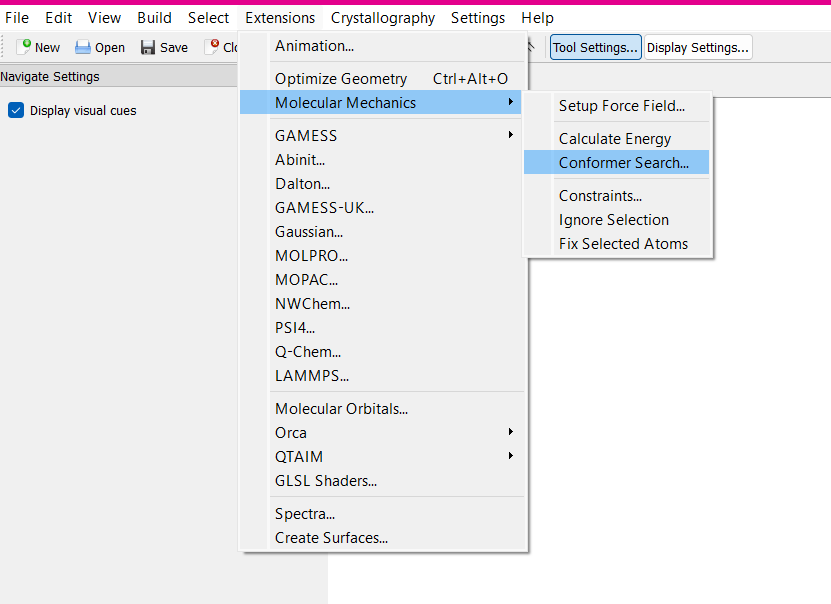

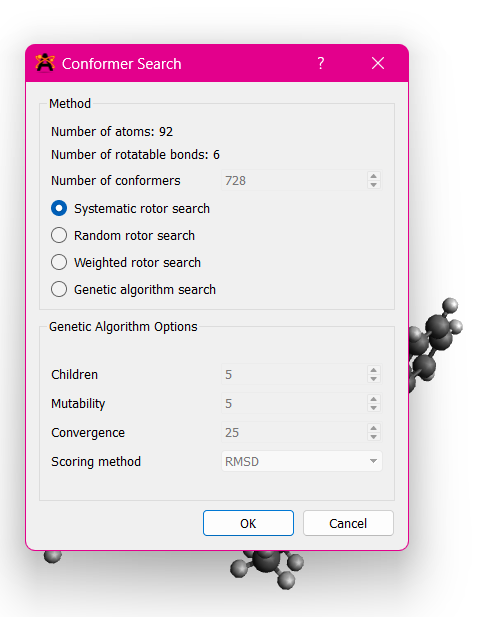
To ensure that your structure has the best conformation, on the main taskbar, select **Extensions > Molecular Mechanics > Conformer Search > Systematic Rotor Search > OK**;
2. After setting up the best structure conformation, on the main taskbar, select **Extensions > Optimize Geometry** to ensure the molecule has the most stable conformation;


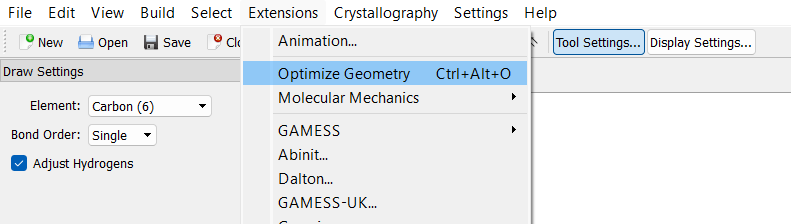


1.
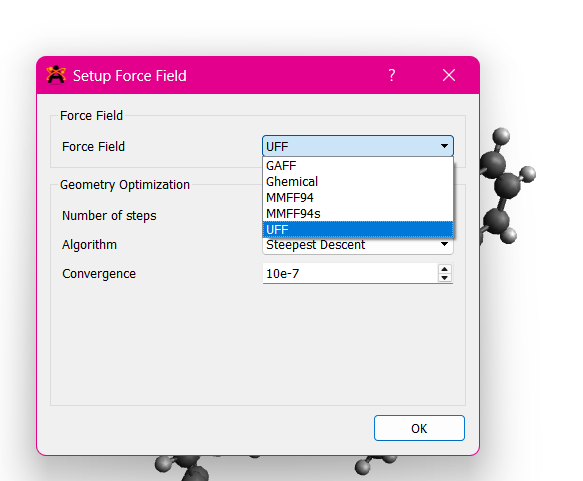

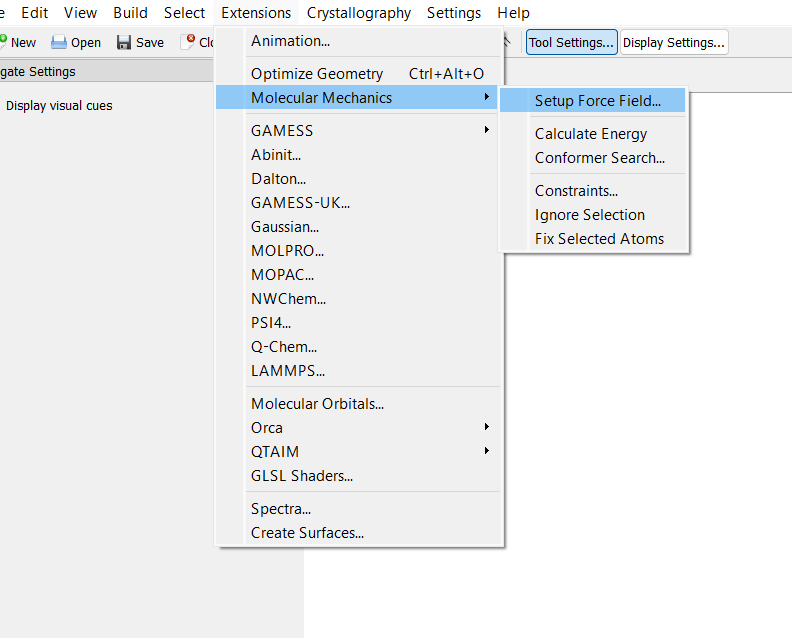
Choose the appropriate force field for your molecule. UFF is suitable for a wide range of elements and molecules, while MMFF94 is often preferred for organic compounds. On the main taskbar, select **Extensions > Molecular Mechanics > Setup Force Field > Force Field: UFF > Steps: 500 > Algorithm: Steepest Descent > OK**;
2.
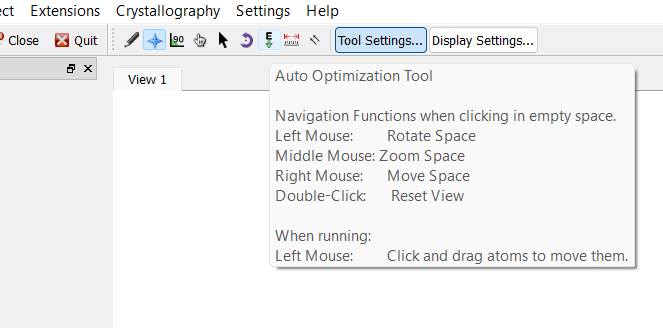
For optimization directly from software and working as an extra step to ensure lowest energy and minimize all interactions, on the main taskbar, select **AutoOptimization tool (letter E with an arrow pointing down) > Force Field: UFF > Steps per Update: 20 > Algorithm: Steepest Descent > OK > Select STOP when molecules are stable.**


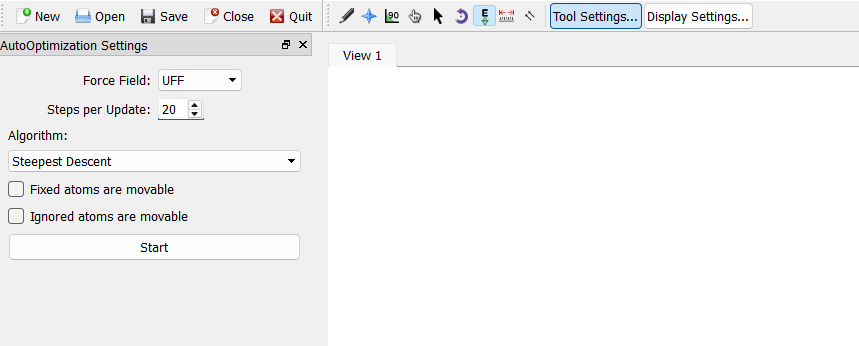


1. To check energy values, on the main taskbar, select **Extensions > Molecular Mechanics > Calculate Energy > Annotate energy value in J.**

-
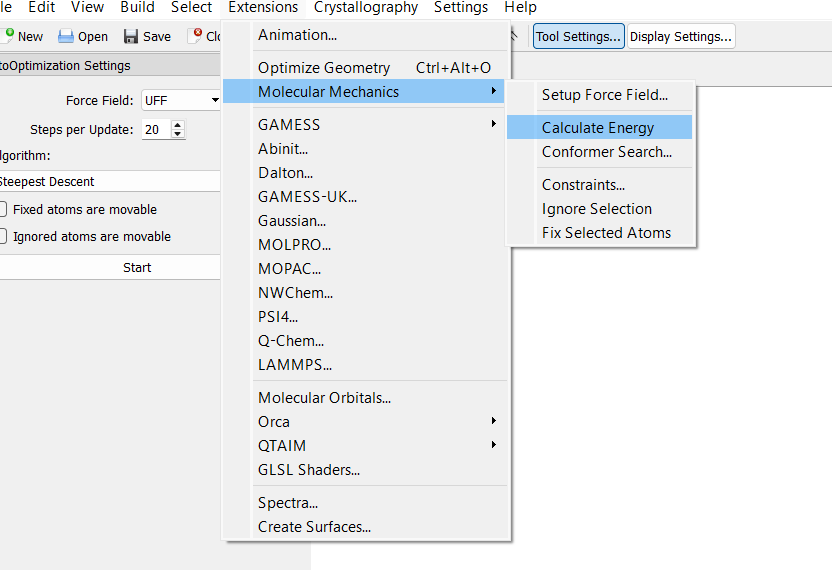
*Once these steps are followed, your ligand is optimized for more accurate binding.*


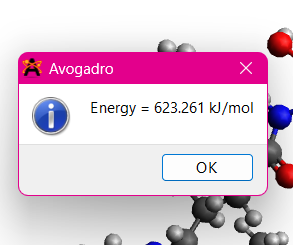


# **2. Protocol for AutoDock Vina Docking using Discovery Studio and PyRx**

## Preparing protein and ligand on Discovery Studio

1. Open the Discovery Studio software.
2. To open the protein structure, on the superior tab, select FILE > OPEN and select the file *“1hsg.pdb”* in the folder where you saved the downloaded protein.

- To change background color and make it easier to see, right-click with the mouse on the background area, and select COLOR > BACKGROUND to choose your preferred color.


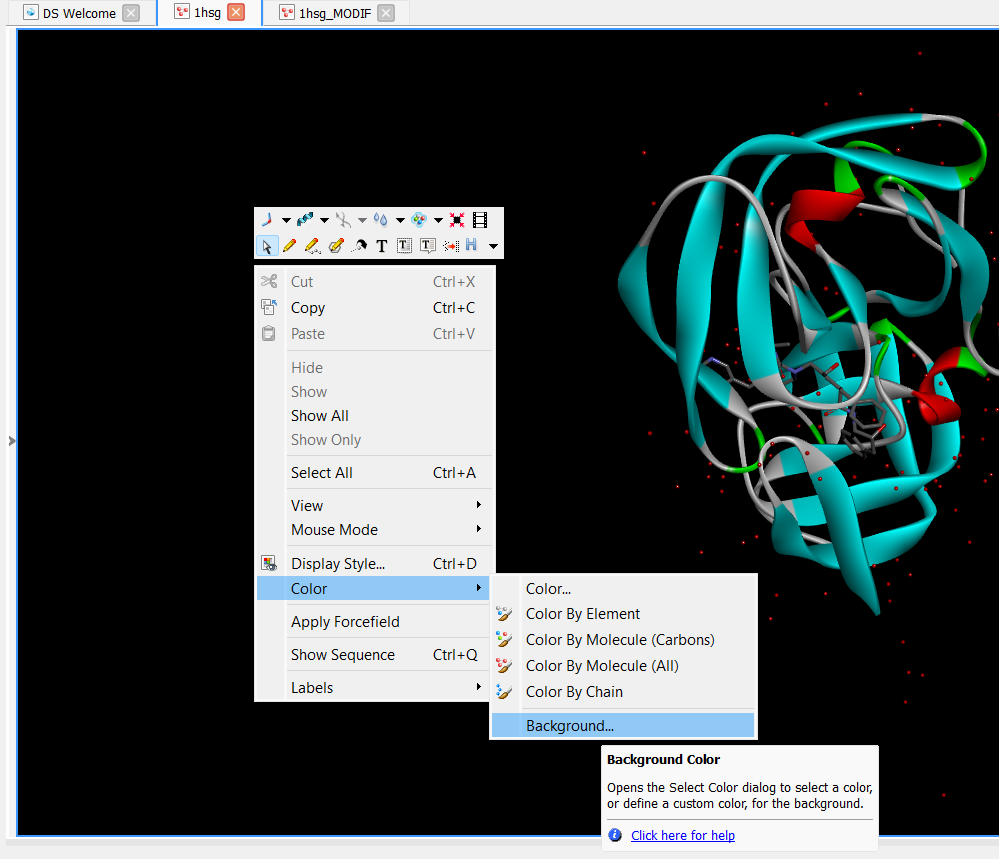


1. To move the protein on the screen, middle click with the mouse and drag it around.
2. To rotate the structure, right click with the mouse and drag it around.
3. To select water molecules, on the superior tab, select SCRIPTS > SELECTION > SELECT WATER MOLECULES.
4. To remove selected molecules, on the superior tab, select EDIT > DELETE or click *“Delete”* on the keyboard.


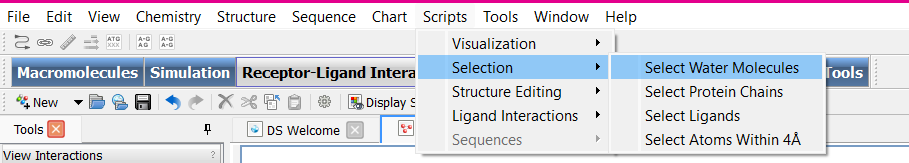


1. The selected water molecules are removed from the structure for this tutorial example.

- Water handling should be decided case by case during receptor preparation. In this tutorial example, water molecules are removed to simplify the redocking workflow.
- On PDB 1HSG, the protein is complexed with Indinavir ligand (structure below), therefore, it is necessary to remove this co-crystallized ligand and save it in .pdb format for redocking.


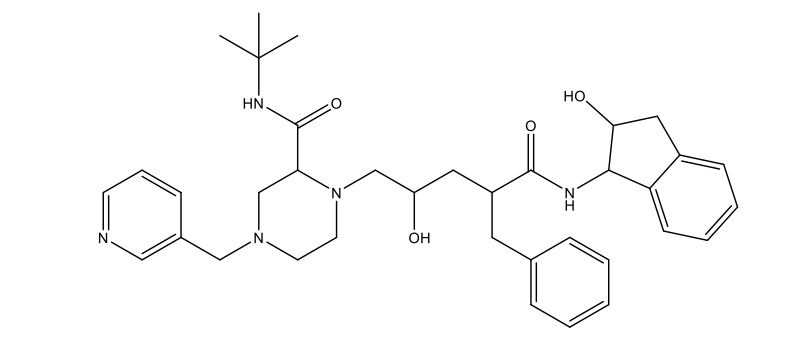


1. To select ligand, on the superior tab, select SCRIPTS > SELECTION > SELECT LIGANDS.
2. Then cut the ligand from the complex with protease, on the superior tab, select EDIT > CUT or click *CTRL + X* on the keyboard.
3. To open a new window, on the superior tab, select FILE > NEW > MOLECULE WINDOW.


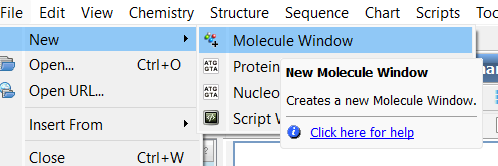


1. Then paste the structure, on the superior tab, select EDIT > PASTE or use CTRL + V on the keyboard.


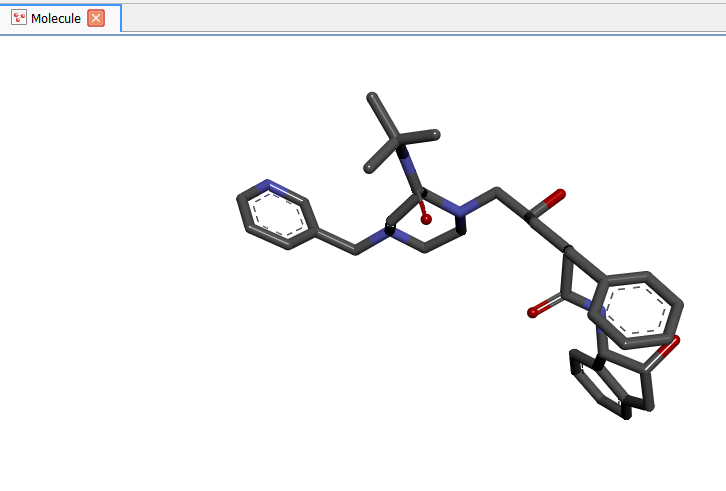


1. Save ligand on .pdb format, on the superior tab, select FILE > SAVE AS; in *“SAVE AS TYPE”,* choose option *“Protein Data Bank Files”* and name the file *“Ligand_1HSG”*.
2. Go back to the protein window by clicking on *“1HSG”* icon on the window’s menu.

To save the protein in .pdb format, follow the steps described in m) and name the file *“1hsg_modif”.*

## **3. PyRx docking**

1. Open PyRx software.

- To change background color, click on *“Configure the scene”* which is the gear icon on the submenu 3D scene window.


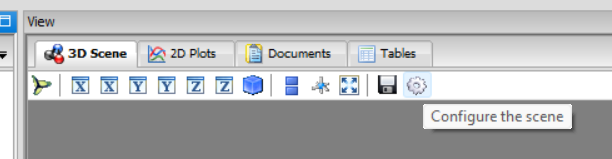


1. On the left side of the superior tab, select FILE > LOAD MOLECULE and select prepared protein file *“1hsg_modif.pdb”.*
2. As mentioned in b), select the prepared ligand *“Ligand_1HSG.pdb”.*
3. On the left menu, left click on *1hsg_modif*, then right click on it, AUTODOCK > MAKE MACROMOLECULE.
4. As mentioned in d), AUTODOCK > MAKE LIGAND.


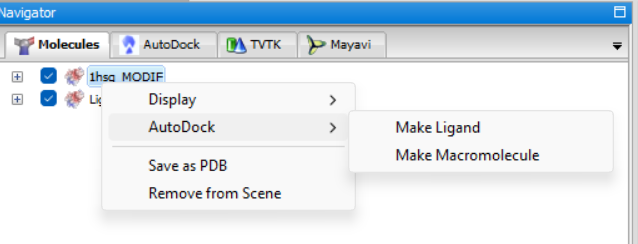


- The last couple of steps convert both structures from *.pdb format* to. pdbqt *format*, which is necessary for docking.

1. Below, on the inferior tab named *“Controls”,* select VINA WIZARD.
2. On VINA WIZARD submenu, select START HERE > LOCAL (located on *Vina Execution Mode*) > START.


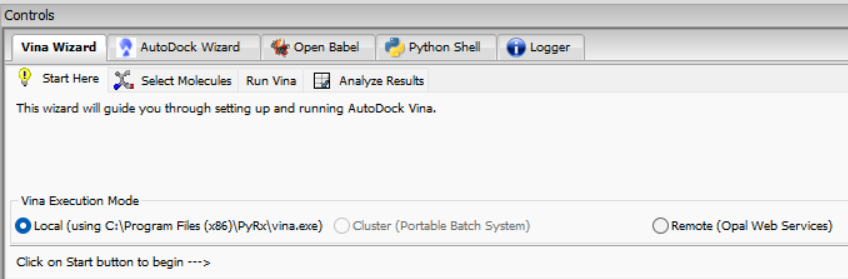


1. To select molecules that are going to be used on this molecular docking study, click once on the ligand and the protein, now located on the left tab, then click FORWARD (located on the left inferior tab).
2. Next step, select the GRID box whose dimensions will represent the search space of the active site, which makes it possible to use a ligand, or one or more amino acid important residues, among others as reference.

- For this tutorial, the amino acid residue used is *“ASP25”* in the *A chain*, which is important for the inhibitory effect in this protein.

1. On *“Molecules”* left menu, click the plus (+) symbol beside protein name, and then again, the plus (+) symbol next to “*A”* (A chain, according to information on protein source).
2. Right click with mouse over residue “*ASP 25”*, select DISPLAY > LABEL > ATOMS to highlight this residue on the protein.


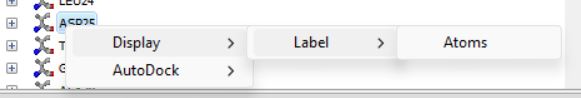


1. A box will show up on screen, that should be positioned centralizing active site, which is “*ASP25”*.

- It is possible to augment or reduce the size of the box through the white circles of the box.

1. Click on FORWARD and wait for calculations.
2. On the left menu, select AUTODOCK tab. The docking scores containing **binding affinity, mode, and RMSD (root mean square deviation),** are shown on the inferior tab.

- When selecting each result, it is possible to observe the different conformations or poses of the ligand.


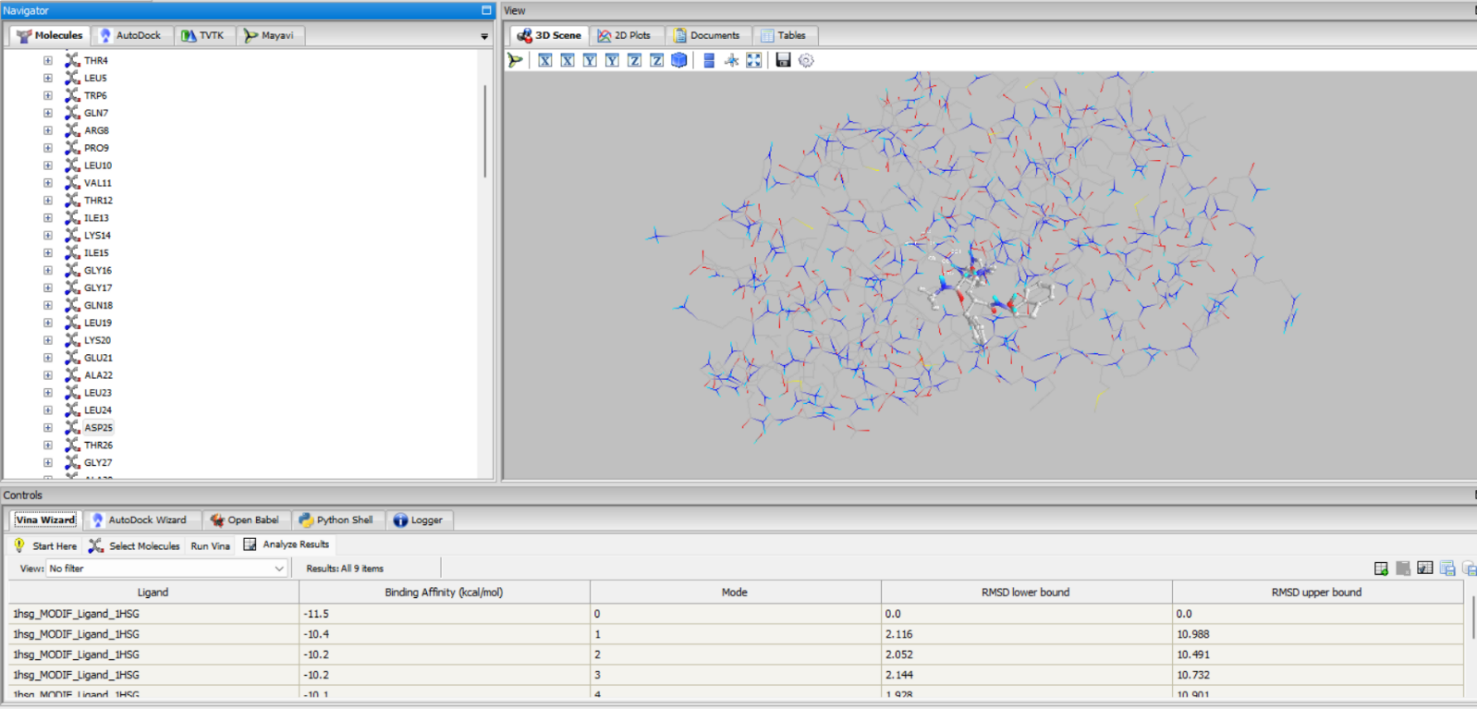


1. On the left menu, select AUTODOCK > Ligand_1HSG_out.pdbqt and observe how every solution shows up superimposed.
2. To be able to visualize protein-ligand interactions, files must be saved. To save the protein, MOLECULES > right click on “*1hsg_modif”* > SAVE AS PDB. Name it *“1hsg_modif”.*
3. As mentioned in n), save ligands by right clicking on *“1hsg_modif_Ligad_1HSG”* and name it *“Model1”.*


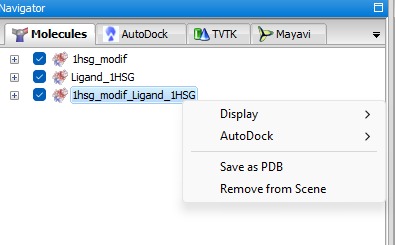


## **4. Complex visualization on Discovery Studio**

1. To visualize the complexes, it is necessary to import the prepared protein and docked ligands into Discovery Studio.
2. Click on OPEN > FILE and load the modified protein.
3. Click on FILE > INSERT FROM and load the complexes.


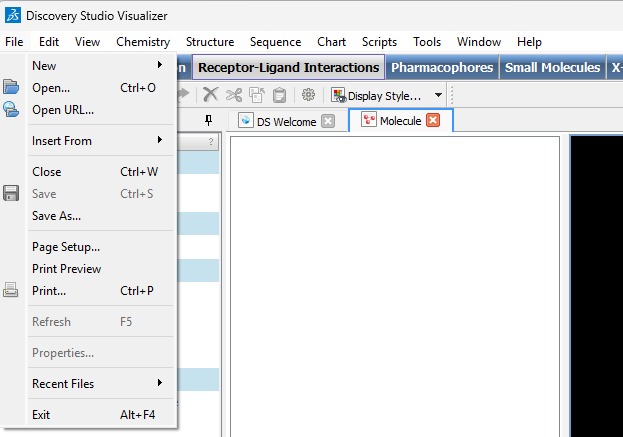


1. On the left menu, select LIGAND INTERACTIONS, and then, SHOW 2D DIAGRAM.


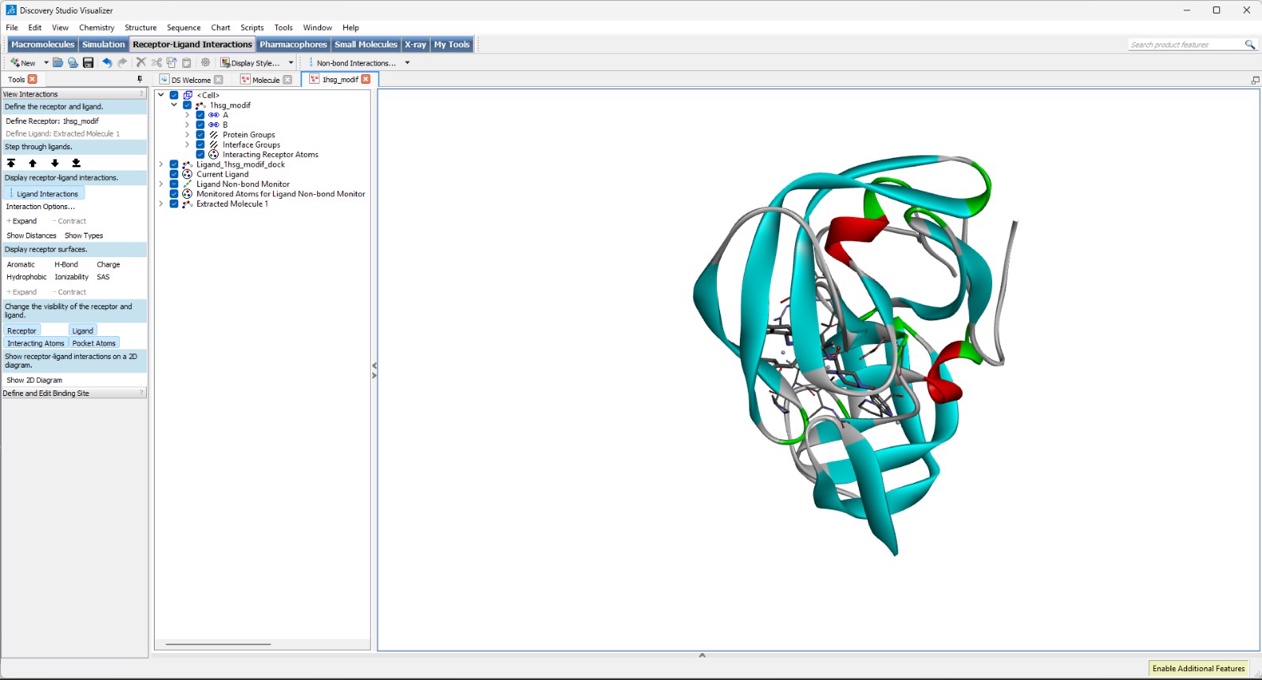


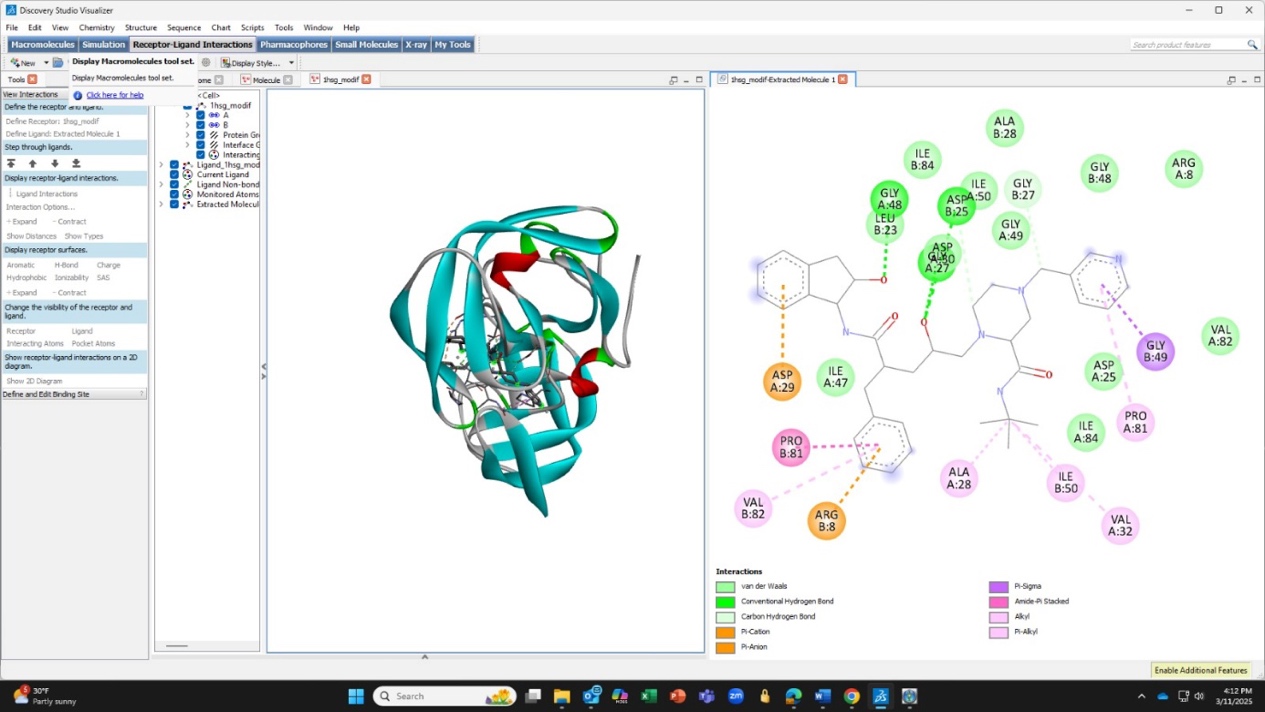


1. To save the 2D image, select FILE > SAVE AS > IMAGE FILES.

The interactions between the receptor and ligands are shown to you to analyze. It is interesting to compare the obtained interactions through redocking with the observed interactions in the crystal and calculate the RMSD.

1. To get RMSD data, open both ligands (from the original structure and docked), select STRUCTURE > RMSD > Heavy atoms, and the results in Å will appear.
2. RMSD values below 2 Å are commonly used as a useful initial indicator of pose recovery in self-docking, but they are not sufficient on their own to establish overall workflow reliability, screening utility, or deployment robustness.
